# *σ*^N^-dependent control of acid resistance and the locus of enterocyte effacement in enterohemorrhagic *Escherichia coli* is activated by acetyl phosphate in a manner requiring flagellar regulator FlhDC and the *σ*^S^ antagonist FliZ

**DOI:** 10.1002/mbo3.183

**Published:** 2014-06-16

**Authors:** Avishek Mitra, Pamela A Fay, Khoury W Vendura, Zimrisha Alla, Ronan K Carroll, Lindsey N Shaw, James T Riordan

**Affiliations:** 1 Department of Cell Biology, Microbiology and Molecular Biology, University of South FloridaTampa, Florida, 33620

**Keywords:** acid resistance, EHEC O157, H7, LEE, NtrC, *rpoN*, sigma factor N

## Abstract

In enterohemorrhagic *Escherichia coli* (EHEC), sigma factor N (*σ*^N^) regulates glutamate-dependent acid resistance (GDAR) and the locus of enterocyte effacement (LEE); discrete genetic systems that are required for transmission and virulence of this intestinal pathogen. Regulation of these systems requires nitrogen regulatory protein C, NtrC, and is a consequence of NtrC-*σ*^N^-dependent reduction in the activity of sigma factor S (*σ*^S^). This study elucidates pathway components and stimuli for *σ*^N^-directed regulation of GDAR and the LEE in EHEC. Deletion of *fliZ*, the product of which reduces *σ*^S^ activity, phenocopied *rpoN* (*σ*^N^) and *ntrC* null strains for GDAR and LEE control, acid resistance, and adherence. Upregulation of *fliZ* by NtrC-*σ*^N^ was shown to be indirect and required an intact flagellar regulator *flhDC*. Activation of *flhDC* by NtrC-*σ*^N^ and FlhDC-dependent regulation of GDAR and the LEE was dependent on *σ*^N^-promoter *flhD*_P__2_, and a newly described NtrC upstream activator sequence. Addition of ammonium chloride significantly altered expression of GDAR and LEE, acid resistance, and adherence, independently of *rpoN*, *ntrC*, and the NtrC sensor kinase, *ntrB*. Altering the availability of NtrC phosphodonor acetyl phosphate by growth without glucose, with acetate addition, or by deletion of acetate kinase *ackA*, abrogated NtrC-*σ*^N^-dependent control of *flhDC*, *fliZ*, GDAR, and the LEE.

## Introduction

Alternative sigma factor N (*σ*^N^) when bound to RNA polymerase directs the transcription of genes for carbon and nitrogen metabolism, stress fitness, and regulation (Reitzer and Schneider 2001). In an increasing number of bacterial pathogens, *σ*^N^ also regulates genes for virulence and transmission, and is required for complete in vivo disease progression (Okada et al. 2008; Barchiesi et al. 2009; Albert-Weissenberger et al. 2010; Damron et al. 2012; Iyer and Hancock 2012; Mills et al. 2012; Sheng et al. 2012; Wang et al. 2012). For most pathogens, the mechanism underlying *σ*^N^-dependent regulation of pathogenesis remains unknown; two exceptions to this include *Borrelia burgdorferi*, and to a lesser extent, enterohemorrhagic *Escherichia coli* (EHEC). In *B. burgdorferi*, the causative agent of Lyme borreliosis, *σ*^N^ activates the expression of genes encoding outer surface lipoproteins (OspA and OspC) essential for transmission from the tick vector to a mammalian host, and for establishment of infection (Pal et al. 2000; Hubner et al. 2001; Grimm et al. 2004). This Osp activation pathway requires another sigma factor, *σ*^S^, the transcription of which is directly activated from a *σ*^N^-promoter in what has been dubbed a *σ*^N^-*σ*^S^ regulatory cascade (Smith et al. 2007; He et al. 2008). In EHEC serotype O157:H7, a food-borne pathogen attributed to outbreaks and sporadic cases of bloody diarrhea (hemorrhagic colitis) (Rangel et al. 2005), *σ*^N^ (encoded by *rpoN*) represses transcription of glutamate-dependent acid resistance (GDAR) genes, while activating the locus of enterocyte effacement (LEE) pathogenicity island (Riordan et al. 2010). The GDAR system allows for low oral infectious dose during gastric passage (Chart 2000; Teunis et al. 2004), while the LEE encodes a type III secretion (T3S) apparatus that translocates virulence factors into host intestinal cells mediating intimate adherence and immune subversion (McDaniel and Kaper 1997; Elliott et al. 1998; Perna et al. 1998). Thus, *σ*^N^ in EHEC regulates major determinants of fecal–oral transmission and colonization.

Like *B. burgdorferi*, a *σ*^N^-*σ*^S^ regulatory pathway has been described for EHEC, and has been further implicated in the control of GDAR and LEE genes in this pathogen (Riordan et al. 2010). However for EHEC, the underlying mechanism by which *σ*^S^ is regulated is not completely understood. *σ*^S^ controls the expression of hundreds of genes in *E. coli* (Hengge-Aronis 2002); it is an activator of GDAR system genes (*gad* genes) (Ma et al. 2003), and can both activate and repress the LEE (Iyoda and Watanabe 2005; Tomoyasu et al. 2005; Laaberki et al. 2006). Strains null for *rpoN* are characterized by a phenotype of increased GDAR and decreased LEE expression that is dependent on an intact *rpoS* (encoding *σ*^S^), and while deletion of *rpoN* in either EHEC or laboratory *E. coli* strain K-12 MG1655 has no impact on *rpoS* transcription, both the stability and activity of *σ*^S^ have been shown to increase (Riordan et al. 2010; Dong et al. 2011; Mitra et al. 2012). Mitra et al. (2012) demonstrated that this effect of *σ*^N^ on *σ*^S^ stability/activity is indirect and dependent on transcription from a *σ*^N^ promoter, and not competition of these sigma factors for core RNA polymerase (RNAP). What additional regulatory component(s) is required downstream of *σ*^N^ for control of *σ*^S^, GDAR, and the LEE is not yet known. Unlike other *E. coli* sigma factors, the initiation of transcription by *σ*^N^ requires activation by enhancer-binding proteins (EBP) that communicate various environmental signals to the RNAP-*σ*^N^ holoenzyme complex (E*σ*^N^) (Shingler 1996). Of the 11 EBPs encoded within the EHEC O157:H7 background, only deletion of *ntrC* (encoding NtrC) phenotypically reproduces the *rpoN* null background for control of *σ*^S^, GDAR, and the LEE (Mitra et al. 2012). Nitrogen regulatory protein C, NtrC (also NRI), is the response regulator of a two-component system that activates *σ*^N^-dependent transcription of genes for the assimilation and utilization of nitrogen, relieving slowed growth under nitrogen-limiting conditions (Zimmer et al. 2000). It is thus plausible that nitrogen availability plays a fundamental role in activation of the *σ*^N^-*σ*^S^ regulatory pathway in EHEC. The objective of this study was to identify additional regulatory factors required for *σ*^N^-dependent control of *σ*^S^, acid resistance, and the LEE, and to examine the role for nitrogen availability in the stimulation of this pathway. The study identifies *flhD* and *fliZ* as new genetic determinants of this pathway and provides evidence that NtrC-*σ*^N^-FlhDC-dependent activation of *fliZ*, the product of which modulates *σ*^S^ activity, is needed for regulation of GDAR and the LEE. Furthermore, the availability of acetyl phosphate, not ammonia, is shown to be an important factor for pathway activation.

## Experimental Procedures

### Bacterial strains and growth conditions

All strains and plasmids used in this study are listed in Table1. Luria-Bertani (LB) starter cultures were inoculated with a single colony of each strain and grown at 37°C with shaking (200 rpm) to an optical density at 600 nm (OD_600_) of 0.5. Unless otherwise indicated, these cultures were used to inoculate either Dulbecco's Modified Eagle's Medium (DMEM) (Sigma-Aldrich, St. Louis, MO) buffered with 50 mmol/L 3-morpholinopropane-1-sulfonic acid (MOPS) and containing 0.4% (w/v) glucose, or MOPS minimal medium. MOPS medium was prepared as described in Neidhardt et al.'s (1974) study, and contained 0.4% (w/v) glucose, 0.1% (w/v) NH_4_Cl, and 0.1% (w/v) l-glutamine. Cultures were grown for 18–20 h before inoculating into fresh DMEM or MOPS to a final OD_600_ = 0.05, respectively, using a 1:10 ratio of media-to-flask volume and grown at 37°C, 200 rpm. Appropriate antibiotics were added to cultures as required.

**Table 1:** Strains and plasmids used in this study.

Strain/Plasmid	Relevant characteristics	Source/Reference
Strain name		
DH5*α*	Vector propagation, *recA1 endA1*	
BL-21		Miroux and Walker (1996)
TW14359	WT O157:H7 2006 outbreak, western US (NC_013008.1)	Manning et al. (2008)
EcRPF-6	TW14359Δ*rpoN*	Mitra et al. (2012)
EcRAM-26	TW14359Δ*ntrC*	Mitra et al. (2012)
EcRAM-43	TW14359Δ*rpoN*Gln+, suppressor mutant for Gln auxotrophy	This study
EcRAM-45	EcRAM-43Δ*glnA*	This study
EcRAM-47	TW14359*crl*::*kan* Kan^R^	This study
EcRAM-49	TW14359Δ*fliZ*	This study
EcRAM-51	EcRFP-6 pRAM-3 Amp^R^	This study
EcRAM-52	EcRAM 26 pRAM-3 Amp^R^	This study
EcRAM-53	EcRAM 49 pRAM-3 Amp^R^	This study
EcRAM-58	TW14359Δ*flhDC*	This study
EcRAM-59	EcRAM 58 pRAM-4 Amp^R^	This study
EcRAM-60	EcRAM 58 pRAM-5 Amp^R^	This study
EcRAM-61	EcRAM 58 pRAM-6 AmpR	This study
EcRAM-63	TW14359Δ*ackA*	This study
EcRAM-66	TW14359Δ*fliZ*pRAM-8	This study
EcRAM-68	TW14359Δ*ackA*pRAM-9	This study
Plasmid name		
pACYC177	Low-copy cloning vector, Amp^R^ Kan^R^ P15A	Chang and Cohen (1978)
pET-24d	IPTG-inducible His-tagging vector, Kan^R^	Novagen
pBAD22	Mid-copy arabinose-inducible cloning vector, Amp^R^	Guzman et al. (1995)
pSC-B	High-copy cloning vector, Amp^R^ Kan^R^	StrataClone
pBAD-TA	Mid-copy arabinose-inducible cloning vector, Amp^R^	Invitrogen
pRAM-1	*rpoN*::pACYC177, Amp^R^ Kan^S^	Mitra et al. (2012)
pRAM-3	*flhDC*::pBAD22, Amp^R^	This study
pRAM-4	*flhDC*::pACYC177 positions +948 to −1994 relative to start codon	This study
pRAM-5	*flhDC*::pACYC177 positions +948 to −825 relative to start codon	This study
pRAM-6	*flhDC*::pACYC177 positions +948 to −728 relative to start codon	This study
pRAM-7	*ntrC*::pET-24d containing ORF, Kan^R^	This study
pRAM-8	*fliZ*::pBAD, Amp^R^	This study
pRAM-9	*ackA*::pSC-B, Amp^R^ Kan^R^	This study

### Procedures for genetic manipulation

Nonpolar gene deletion mutants were constructed using the *λ* Red recombinase-assisted approach (Datsenko and Wanner 2000; Murphy and Campellone 2003) and as described previously (Riordan et al. 2010). Primers used for the construction of deletion mutants are listed in Table S1. For overexpression of *flhDC*, a 932-bp polymerase chain reaction (PCR) fragment containing *flhDC* of strain TW14359 (nucleotide positions 2485400–2484469) was generated using primers flhDC-F/EcoRI and flhDC-R/XbaI. An *Eco*RI/*Xba*I digested fragment of the product was cloned into similarly digested arabinose-inducible expression vector pBAD22 (Guzman et al. 1995) to produce pRAM-3. pRAM-3 purified from DH5*α* transformants was then used to transform TW14359 and derivative strains producing EcRAM-51 through EcRAM-53. For *flhDC* promoter expression studies, a 2942-bp *Xho*I/*Bam*HI digested PCR fragment (nucleotide positions 2487394–2484453) was generated using primers flhD-1994/XhoI and flhC+595/BamHI. This fragment contained the *flhDC* open reading frames (ORFs) and 1994 bp of DNA upstream of the *flhD* start codon including a *σ*^N^ promoter (2486152–2486138), a *σ*^70^ promoter (2485633–2485604), and a predicted NtrC box (2487152–2487132). This was ligated into *Xho*I/*Bam*HI digested pACYC177 to produce pRAM-4. The same approach was used for pRAM-5 and pRAM-6 construction, however, the cloned fragment in pRAM-5, generated using primers flhD-825/XhoI and flhD+595/BamHI (positions 2486228-2484453), did not include the predicted NtrC box. For the fragment in pRAM-6, generated using primers flhD-728/XhoI and flhD+595/BamHI (positions 2486128–2484453), both the NtrC box and the *σ*^N^ promoter were excluded. Plasmids were purified from DH5*α* transformants and used to transform TW14359Δ*flhDC* producing strains EcRAM-59 to EcRAM-61. For *fliZ* complementation, a 552-bp PCR fragment containing the *fliZ* ORF was created using primers fliZ-Clone/F and fliZ-Clone/R and cloned into the arabinose-inducible pBAD-TA vector (Invitrogen, Grand Island, NY) to yield pRAM-8, which was then used to transform EcRAM-49 to produce EcRAM-66. For *ackA* complementation, the *ackA* ORF was amplified using primers ackA-Clone/F and ackA-Clone/R and cloned into the high copy pSC-B vector (Agilent, Santa Clara, CA) to create pRAM-9, which was then used to transform EcRAM-63 to produce EcRAM-68. The *rpoN* complement strain EcRAM-36 was constructed previously (Mitra et al. 2012). All genetic constructs were validated using a combination of restriction mapping, DNA sequencing, and quantitative real-time PCR (qRT-PCR).

### Quantitative real-time PCR

RNA purification, cDNA synthesis, qRT-PCR cycling conditions, and data analysis for relative quantitation of gene expression followed previously described protocols (Riordan et al. 2010; Mitra et al. 2012; Morgan et al. 2013). Analysis was performed using a Realplex2 Mastercycler (Eppendorf, Hauppauge, NY). Cycle threshold (C_t_) data were normalized to *rrsA* (16S rRNA gene) and normalized C_t_ values (ΔC_t_) were transformed to arbitrary gene expression units using 2^−ΔCt^/10^−6^ as described by Livak and Schmittgen (2001). A previous method was used for the quantitation of *flhD* mRNA copy number (Bustin 2000). Briefly, a 154-bp PCR product containing *flhD* was generated using flhD+63 and flhD+216, column purified (Qiagen, Valencia, CA) and serially diluted in molecular grade water. C_t_ was measured for each dilution to generate a standard curve plotting C_t_ as a linear function of DNA concentration (ng/*μ*L). The strength of linearity was estimated by the correlation coefficient (*r*^2^), which exceeded 0.90 for all curves. DNA concentration was extrapolated from a standard curve using experimental C_t_ values and then converted to *flhD* copy number based on the estimated weight of a single 154-bp *flhD* dsDNA fragment of 47-kDa. Gene expression levels and *flhD* copy number were compared between samples using the appropriate *t*-test or by analysis of variance (ANOVA) and Tukey's HSD (*n* ≥ 3, *α* = 0.05) using R v. 2.13.0.

### Protein extraction, sodium dodecyl sulfate polyacrylamide gel electrophoresis, and western blots

Protein extraction, purification, and procedures for western blots followed a previously described protocol (Mitra et al. 2012; Morgan et al. 2013). Monoclonal antibodies for *σ*^S^ and GroEL were acquired from Neoclone (Madison, WI) and Bio-Rad (Carlsbad, CA), respectively. Densitometry was used to estimate differences in protein levels using a ChemiDoc XRS+ Imaging System and Image Lab 3.0 (Bio-Rad, Hercules, CA). Western blots were repeated a minimum of three times in independent trials.

### Purification of NtrC

A 1425-bp *Nco*I/*Xho*I-digested PCR fragment generated using primers ntrC+F/NcoI and ntrC-R/XhoI was cloned into similarly digested pET-24d producing pRAM7 and replacing the *ntrC* stop codon with a C-terminal 6xHis tag. pRAM7 was transformed into propagating *E. coli* strain BL-21, which was grown in LB containing ampicillin (100 *μ*g/mL) to OD_600_ = 0.4 before induction of 6xHis-tagged *ntrC* with 1 mmol/L Isopropyl *β*-D-1-thiogalactopyranoside for 16 h at 20°C (200 rpm). Cultures were harvested by centrifugation (5000*g*, 20 min) and 6xHis-NtrC was purified using a nickel Ni-NTA Protein Purification Kit (Qiagen) according to the manufacturer's instruction.

### Electrophoretic mobility shift assay

Electrophoretic mobility shift assay (EMSA) was performed using the LightShift Chemiluminescence EMSA Kit (Pierce, Rockford, IL) according to the manufacturer's instruction. Biotin end-labeled DNA probes were generated by PCR using flhD-1842/Biotin and flhD-1634/Biotin for the *flhD*_P_ promoter probe, and glnA-311/Biotin and glnA-112/Biotin for the *glnA*_P2_ promoter probe; biotin end-labeled Epstein–Barr nuclear antigen (EBNA) DNA was supplied with the kit. The *flhD*_P_ promoter probe (strain TW14359 nucleotide position 2487034–2487242) contained a putative NtrC-binding site flanked by 0.1 kb. For the *glnA*_P2_ promoter probe, a confirmed NtrC box (nucleotide position 4913213–4913228) was flanked by 0.1 kb. Binding reactions (20 *μ*L per reaction) contained 20 fmol of biotin end-labeled DNA probe, 50 mmol/L KCl, 5 mmol/L MgCl_2_, 1% (v/v) glycerol, 0.05% (v/v) NP-40, 50 ng/*μ*L poly(dI-dC) copolymer competitor, 10x molar excess Bovine Serum Albumin (10 mg/mL), and 0, 2, 4, 8, or 16 *μ*mol/L purified C-terminally labeled 6xHis-NtrC. Reactions were incubated for 40 min at 4°C, and were then separated by electrophoresis using 8% nondenaturing acrylamide gels prepared in 0.5x Tris-borate-EDTA buffer at 4°C for 80 min at 160 V, and DNA/protein complexes transferred to a nylon membrane (Fisher, Pittsburgh, PA). Membranes were UV cross-linked at 120,000 mJ/cm^2^ for 1 min and detected by chemiluminescence using the Biotin Detection System (Pierce) and a ChemiDoc XRS+ Imaging System including Image Lab 3.0 (Bio-Rad, Hercules, CA).

### Selection of suppressor mutants for glutamine auxotrophy

Spontaneous suppressor mutants for glutamine auxotrophy were selected in the TW14359Δ*rpoN* background by growth in MOPS minimal media without the addition of glutamine. Briefly, overnight cultures of TW14359Δ*rpoN* grown in MOPS media were inoculated into fresh MOPS containing 0.4% glucose and 0.1% NH_4_Cl and grown at 37°C (200 rpm). The outgrowth of suppressor mutants (TW14359Δ*rpoN* Gln+) consistently occurred following 48-h incubation. Single colonies of suppressor mutants were obtained by subculture from MOPS media to LB with 1.5% agar, and confirmed by growth in MOPS containing 0.2% glucose and 0.1% (w/v) l-histidine as described by Reitzer et al. (1987) and by qRT-PCR analysis of glutamine synthetase *glnA* expression. Three independent suppressor mutants were selected and validated by this approach. The mutation leading to suppression was determined using a combination of PCR and Sanger sequencing of amplified DNA fragments (MWG Operon, Huntsville, AL) and next-generation whole genome sequencing.

### Whole genome next-generation DNA sequencing and analysis

Genomic DNA was extracted from TW14359Δ*rpoN* and a single suppressor mutant of TW14359Δ*rpoN* (TW14359Δ*rpoN* Gln+) using Puregene® Kits (Gentra, Minneapolis, MN). One microgram of DNA from each strain was enzymatically sheared into libraries of ∼200-bp fragments using the Ion Xpress™ Plus Fragment Library Kit (Life Technologies, Grand Island, NY). Each DNA library was purified using the E-Gel® SizeSelect™ 2% Agarose system (Invitrogen), and the integrity and quantity of each was determined using a Bioanalyzer high-sensitivity DNA chip (Agilent). Libraries were diluted and template-positive Ion Sphere Particles (ISPs) prepared using the Ion OneTouch 200 Template Kit (Life Technologies). ISPs were sequenced using an IonTorrent™ Personal Genome Machine and the Ion PGM 200 Sequencing Kit (Life Technologies) following the manufacturer's instructions. Whole genome sequencing data were exported from the Ion Torrent Server and analyzed using the Genomics Suite software package (CLC Bio, Boston, MA). Genomes were assembled using the TW14359 genome (NC_013008, NCBI) as a reference, followed by quality-based variant detection to identify polymorphisms with a minimum coverage of 10x and 100% detection frequency. Polymorphisms common to both strains (relative to the reference TW14359 genome), and those in homopolymeric nucleotide tracts, were excluded resulting in the identification of specific genetic variations between TW14359Δ*rpoN* and TW14359Δ*rpoN* Gln+.

### Adherence assay

Adherence to epithelial cells was determined following a previously described protocol (Morgan et al. 2013). Briefly, human HT-29 colonic epithelial cells were grown to confluence on polylysine-treated glass coverslips placed within the wells of 24-well culture plates at 37°C with 5% CO_2_. Overnight DMEM cultures were diluted 1:40 (v/v) in fresh DMEM and 0.05 mL of this dilution was used to inoculate each well which already contained 0.45 mL of sterile DMEM. After 3 h, plate wells were washed five times with PBS (137 mmol/L NaCl, 2.7 mmol/L KCl, 10 mmol/L Na_2_HPO_4_, pH 7) to remove nonadherent bacteria from the coverslips, and fresh DMEM was then added before incubating for an additional 3 h. Plate wells were subsequently washed three times in PBS, and then fixed with ice cold (−20°C) 100% methanol for 10 min before staining with Giemsa diluted in PBS 1:20 (v/v) for 20 min. Giemsa-stained coverslips were examined at 1000× magnification by oil immersion, and microcolonies were scored as discrete clusters of five or more bacterial cells as previously defined (McKee and O'Brien 1995; Abe et al. 2002; Iyoda and Watanabe 2004). For each sample, a minimum of 10 viewing frames were observed and the average number of microcolonies were reported per 50 HT-29 cells. Microcolony counts were compared between strains by Tukey's HSD following a significant *F*-test (*n* ≥ 3, *α *= 0.05) (R v. 2.13.0).

### Tests for acid resistance

Acid resistance by the glutamate-dependent system was measured for exponential phase cultures grown in DMEM as previously described (Riordan et al. 2010; Mitra et al. 2012) with slight adaptations. Strains were grown in DMEM to OD_600_ = 0.5 before inoculating (10^6^ CFU/mL final cell density) into E minimal glucose (EG) media containing 5.7 mmol/L l-glutamate adjusted with HCl to pH 7 (control) or pH 2. Cultures were sampled for counts (CFU/mL) after 1 h incubation at 37°C (200 rpm) by plating serial dilutions to LB with 1.5% agar and incubating overnight. Experiments were repeated a minimum of three times in independent trials.

## Results

### NtrC-*σ*^N^ require *fliZ* for control of *σ*^S^ activity, GDAR, and the LEE

**Figure 1:**
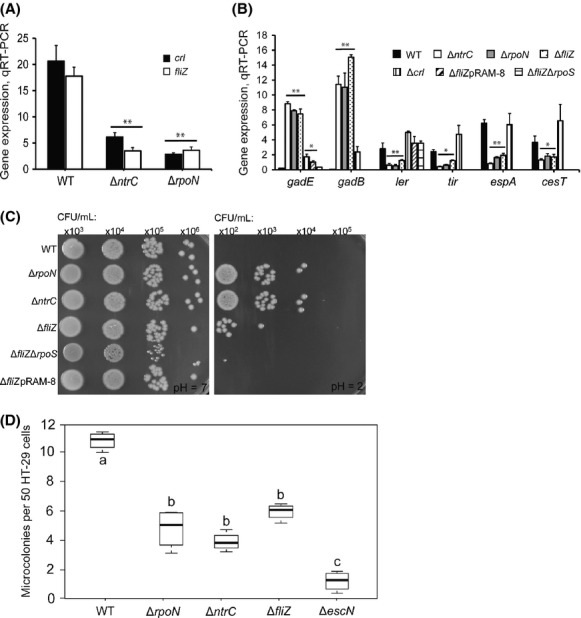
Effect of *fliZ* deletion on GDAR and LEE expression, acid resistance, and adherence. (A) Expression of *crl* (black) and *fliZ* (white) plotted for wild-type and derivative strains. (B) Expression of GDAR (*gadE*, *gadB*) and LEE (*ler*, *tir*, *espA*, *cesT*) genes plotted for wild type (black), Δ*ntrC* (white), Δ*rpoN* (gray), Δ*fliZ* (stippled), and Δ*crl* (vertical lines); in strains Δ*fliZ*pRAM-8 (diagonal) and Δ*fliZ*Δ*rpoS* (dashed), only *gadE* and *ler* were measured. (C) Representative colony-forming units (CFU/mL) on LBA for wild-type and derivative strains following 1-h challenge in EG media (pH 7 vs. pH 2). (D) Counts for microcolonies on HT-29 cells plotted for wild-type and mutant derivative strains. For A and B, asterisks denote significant differences between wild-type and derivative strains by *t*-test (*P < 0.05, **P < 0.01, *n *≥ 3). Error bars denote standard deviation. For D, boxplot boundaries represent the 25th and 75th percentiles, whiskers represent the maximum and minimum values, and the median is given by the horizontal line. Plots that differ in lowercase letter differ significantly by Tukey's HSD following a significant *F*-test (*n* ≥ 3, *P* < 0.05). GDAR, glutamate-dependent acid resistance; LEE, locus of enterocyte effacement; EG, E minimal glucose.

Previous studies have revealed NtrC and *σ*^N^ negatively regulates GDAR and positively regulates the LEE by reducing the activity of alternative sigma factor S (*σ*^S^) (Riordan et al. 2010; Mitra et al. 2012). For this to occur, NtrC-*σ*^N^ must increase or decrease the expression of a gene(s) whose product, in-turn, alters *σ*^S^-dependent transcription. One of two proteins were predicted to fulfill this role: Crl or FliZ. Crl enhances RNAP-*σ*^S^ holoenzyme formation, thus increasing transcription from *σ*^S^ promoters (Pratt and Silhavy 1998; Typas et al. 2007), whereas FliZ interferes with *σ*^S^ promoter-binding and transcription initiation, thus reducing *σ*^S^-dependent transcription (Pesavento et al. 2008; Pesavento and Hengge 2012). During growth in DMEM (OD_600_ = 0.5), both *crl* and *fliZ* expression were shown to be reduced in TW14359Δ*ntrC* and TW14359Δ*rpoN* when compared to TW14359 (*P* < 0.05) (Fig.1A), however, only TW14359Δ*fliZ* phenocopied TW14359Δ*ntrC* and TW14359Δ*rpoN* for the control of GDAR and LEE genes (Fig.1B). In TW14359Δ*crl*, both *gadE* and *gadB* were increased in expression compared to TW14359 (*P* < 0.05), but less than for TW14359Δ*rpoN*, TW14359Δ*ntrC*, and TW14359Δ*fliZ*, in which expression levels for all genes were nearly identical (*P* < 0.01) (Fig.1B). The expression of LEE genes *ler*, *tir*, *espA*, and *cesT* did not differ between TW14359 and TW14359Δ*crl*, but were uniformly reduced in TW14359Δ*ntrC*, TW14359Δ*rpoN*, and TW14359Δ*fliZ* backgrounds (*P* < 0.05) (Fig.1B). Both *gadE* and *ler* expressions were restored to near wild-type levels in *fliZ* complement strain TW14359Δ*fliZ*pRAM-8 or by the deletion of *rpoS* in TW14359Δ*fliZ* (Fig.1B). Consistent with the effect of *fliZ* deletion on *gadE* and *gadB* expression, CFU/mL of TW14359Δ*fliZ* recovered following exposure to acidified (pH 2) EG media for 1 h increased by 10- to 100-fold compared to TW14359, TW14359Δ*fliZ*pRAM-8, and TW14359Δ*fliZ*Δ*rpoS*, yet remained ∼10-fold less than that observed for TW14359Δ*ntrC* and TW14359Δ*rpoN* (Fig.1C). Furthermore, the ability to form microcolonies on HT-29 intestinal cells was decreased in TW14359Δ*fliZ* compared to TW14359 (*P* = 002), and matched that observed for TW14359Δ*ntrC* and TW14359Δ*rpoN* (Fig.1D). Thus, NtrC-*σ*^N^ positively regulate *fliZ* during exponential growth, the product of which is predicted, downregulates GDAR and upregulates the LEE by reducing the activity of extant *σ*^S^.

### Requirement for *flhDC* in the activation of *fliZ* by NtrC-*σ*^N^

*fliZ* is encoded as the second gene of a three gene operon (*fliAZY*), the transcription of which is directed from at least two promoters, *fliA*_P1_ and *fliA*_P2_. Neither of these promoters are *σ*^N^-dependent, however, *fliA*_P1_ is activated by the regulator of flagellar biosynthesis and motility FlhDC, for which there is a predicted *σ*^N^-dependent promoter, *flhD*_P2_ (Zhao et al. 2010). In addition, a putative activator sequence (UAS) for NtrC is present ∼1-kb upstream of *flhD*_P2_. It was thus hypothesized that the control of *fliZ* by NtrC-*σ*^N^ is a consequence of direct activation of *flhDC* transcription from this promoter.

**Figure 2:**
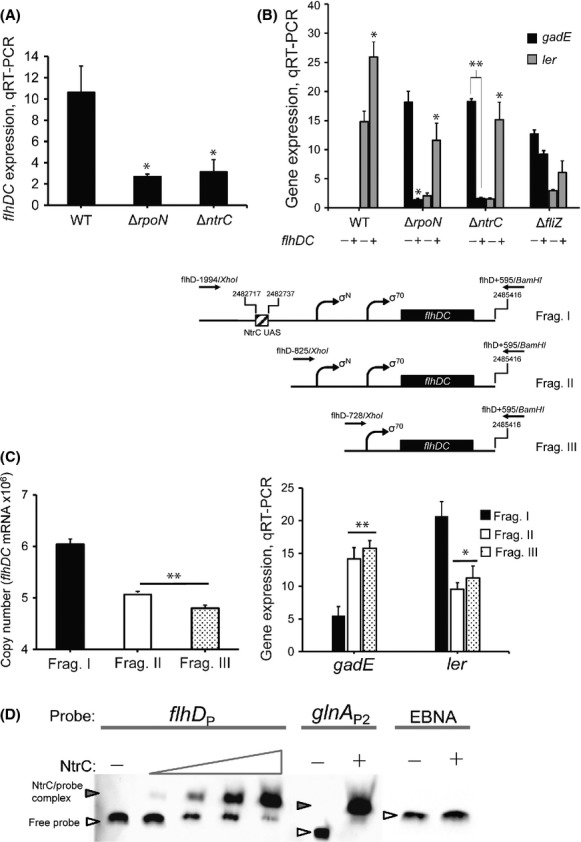
Regulation of *flhDC* by NtrC-*σ*^N^ and effect on *gadE* and *ler* expression. (A) Expression levels for *flhDC* plotted for wild-type and derivative strains. The asterisk denotes a significant difference between wild-type and mutated strains by *t*-test (*P* < 0.05, *n* ≥ 3). (B) Expression levels for *gadE* (black) and *ler* (gray) plotted for wild-type and derivative strains containing pRAM-3 (*flhDC*::pBAD22); expression of pRAM-3 is either uninduced (−) or induced (+) with arabinose. Asterisks indicate significant differences between uninduced and induced treatments by *t*-test (*P < 0.05, **P < 0.01, *n* ≥ 3). (C) Absolute *flhDC* mRNA copy number and expression levels for *gadE* and *ler* measured in the Δ*flhDC* background expressing cloned *flhDC* fragments, Frag. I (black), Frag. II (white), and Frag. III (stippled); topology of *flhDC* promoter fragments are included, top right (C). See text for details. (D) EMSA for NtrC binding to the *flhDC*_P_ promoter and *glnA*_P2_ promoter; EBNA is EBNA DNA. Inset arrows indicate the location of the NtrC/probe complex (filled arrow) or free probe (empty arrow). See text for details. Error bars denote standard deviation for all panels. EMSA, electrophoretic mobility shift assay.

In agreement with this, *flhDC* expression was similarly reduced in both TW14359Δ*ntrC* and TW14359Δ*rpoN* backgrounds compared to TW14359 during growth in DMEM (OD_600_ = 0.5) (*P* < 0.05) (Fig.2A). Also, *flhDC* significantly decreased *gadE* levels and increased *ler* levels when overexpressed in TW14359Δ*ntrC* and TW14359Δ*rpoN* (*P* < 0.05), but not in TW14359Δ*fliZ* (Fig.2B). To define *cis*-elements of the *flhDC* promoter region important for NtrC-*σ*^N^-dependent regulation, *flhDC* mRNA copy number was measured from three promoter fragments (Fig.2C) cloned into arabinose-inducible vector pBAD22 and transformed into TW14359Δ*flhDC*. As anticipated, *flhDC* copy number was reduced when expressed from a fragment in which the putative NtrC UAS was removed (Frag. II) compared to the wild-type *flhDC* promoter fragment (Frag. I) (*P* = 0.004) (Fig.2C). *flhDC* copy number was further reduced when expressed from a fragment in which both the NtrC UAS and putative *σ*^N^ promoter *flhDC*_P2_ were removed (Frag. III), but not significantly less than for Frag. II. Correspondingly, *gadE* expression increased (*P* < 0.01) and *ler* expression decreased (*P* < 0.05) in TW14359Δ*flhDC* expressing either Frag. II or Frag. III when compared to Frag. I (Fig.2C). Thus, the putative NtrC UAS site and *σ*^N^ promoter *flhDC*_P2_ are required for full expression of *flhDC* and for regulation of *gadE* and *ler*. Purified 6xHis-NtrC was observed to retard the mobility by EMSA of a 200-bp *flhD* promoter probe containing the putative NtrC UAS in a manner similar to the NtrC–dependent glutamine synthetase promoter, *glnA*_P2_ (Fig.2D). No shift was observed for *flhD* or *glnA* promoter probes in the absence of 6xHis-NtrC, or for the negative control EBNA DNA probe (Fig.2D).

### Glutamine is not essential for NtrC-*σ*^N^-dependent regulation of GDAR and the LEE

**Figure 3:**
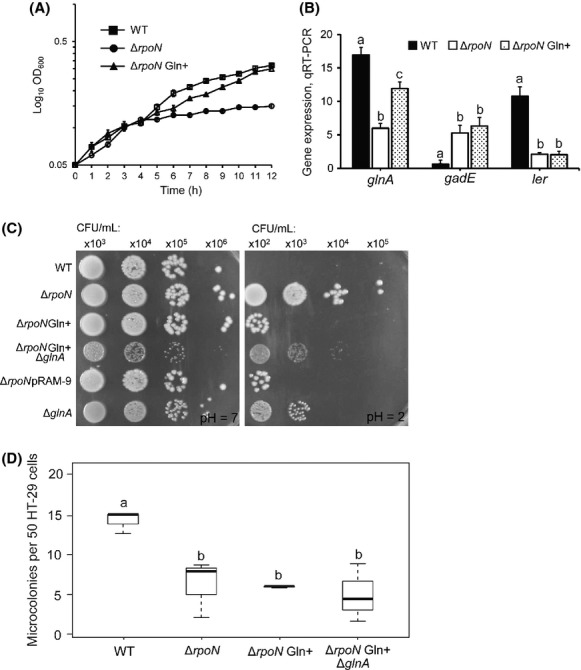
Impact of glutamine metabolism on the GDAR and LEE expression phenotype of TW14359Δ*rpoN*. (A) Mean (*n* = 3) log_10_ transformed optical density at 600 nm (log_10_ OD_600_) plotted for wild type (square), Δ*rpoN* (circles), and suppressor mutant Δ*rpoN*Gln+ (triangles) as a function of time during growth in nitrogen-limiting MOPS media (2 g/L glucose, 1 g/L l-histidine, pH 7). (B) Expression levels for *glnA*, *gadE*, and *ler* plotted for wild type (black), Δ*rpoN* (white), and Δ*rpoN*Gln+ (stippled). Error bars denote standard deviation for A and B. (C) Representative colony-forming units (CFU/mL) on LBA for wild-type and derivative strains following 1-h challenge in EG media (pH 7 vs. pH 2). (D) Counts for microcolonies on HT-29 cells plotted for wild-type and mutant derivative strains. Boxplots are as described for Figure1D. For B and D, plots that differ in lowercase letter for each gene (B) or strain (D) differ significantly by Tukey's HSD following a significant *F*-test (*n* ≥ 3, *P* < 0.05). GDAR, glutamate-dependent acid resistance; LEE, locus of enterocyte effacement.

The preceding experiments reveal NtrC-*σ*^N^ to directly activate *flhDC* transcription, the product of which upregulates *fliZ*. FliZ, in-turn, reduces the activity of *σ*^S^ and consequently, *σ*^S^-dependent control of GDAR and LEE expression. While much is understood as to how *σ*^S^ regulates GDAR and the LEE (Sperandio et al. 1999; Foster 2004; Iyoda and Watanabe 2005; Laaberki et al. 2006), the mechanistic basis for activation of NtrC-*σ*^N^-dependent control of these discrete genetic systems is as yet unknown. NtrC-*σ*^N^ direct the transcription of nitrogen-regulated (Ntr) response genes, the primary function of which is to assimilate nitrogen through induction of transport/scavenging systems and nitrogen degradation pathways (reviewed in Reitzer and Schneider 2001). Under these conditions, glutamine synthetase (GS) catalyzes the synthesis of l-glutamine from ammonia and l-glutamate. The gene for GS (*glnA*) is maximally expressed from the *σ*^N^ promoter *glnA*_P2_ in a manner dependent on NtrC. As such, strains that are null for *rpoN* or *ntrC* cannot initiate transcription from *glnA*_P2_ and are auxotrophic for glutamine when nitrogen is limiting. The significance of glutamine metabolism to NtrC-*σ*^N^-dependent control of GDAR and the LEE was thus examined by selecting a suppressor mutant of glutamine auxotrophy in TW14359Δ*rpoN* and observing its effect on GDAR and LEE gene expression, acid resistance, and adherence. Growth of TW14359Δ*rpoN* in MOPS media containing 0.2% glucose and 0.1% l-histidine (i.e., high energy but nitrogen limiting) is impaired due to auxotrophy for glutamine (Gln−) (Fig.3A). However, after 48 h the outgrowth of a prototrophic (Gln+) suppressor mutant (TW14359Δ*rpoN*Gln+) was repeatedly observed in which wild-type growth in MOPS media was restored (Fig.3A), and in which the expression of *glnA* was significantly increased compared to TW14359Δ*rpoN* during growth in DMEM (OD_600_ = 0.5) (*P* = 0.013) (Fig.3B); *glnA* expression was still, however, slightly but significantly lower in TW14359Δ*rpoN*Gln+ when compared to TW14359 (*P* = 0.02). Mutations which suppress Gln− in *E. coli* have been mapped to *ntrC*, and to *cis*-elements controlling *glnA* transcription. *glnA* can be transcribed from three promoters: *glnA*_P1_ and *glnA*_P3_ are *σ*^70^ promoters that are repressed by NtrC during nitrogen-limitation, whereas *glnA*_P2_ is a *σ*^N^ promoter that is activated by NtrC under the same conditions. Mutations in the DNA-binding domain of NtrC (amino acid residues 400–470) at the C-terminus result in the derepression of *glnA*_P1_ and/or *glnA*_P3_, while mutations in the promoter(s) enhance transcription from *glnA*_P1_ or result in formation of a de novo *σ*^70^ consensus at *glnA*_P2_ (Reitzer et al. 1987). DNA sequencing of *ntrC* and the *glnA* promoter region did not reveal any of these described mutations in TW14359Δ*rpoN*Gln+. Sequencing of the TW14359Δ*rpoN*Gln+ genome, however, revealed a single adenine deletion in the *ntrC* ORF at nucleotide position 4,910,080 (accession NC_013008, NCBI), resulting in a frameshift mutation. This mutation occurs early in the ORF at +285 relative to the start codon and results in a premature stop codon or opal (UGA) mutation at amino acid position 106. It was thus suspected that increased expression of *glnA*, and growth in the absence of glutamine for TW14359Δ*rpoN*Gln+ (Fig.3B), reflects derepression at the *glnA*_P1_ and *glnA*_P3_ promoters due to NtrC inactivation.

Expression levels for *gadE* and *ler* did not differ between TW14359Δ*rpoN* and TW14359Δ*rpoN*Gln+ during growth in DMEM (OD_600_ = 0.5), indicating that glutamine availability has no impact on GDAR and LEE gene regulation. Interestingly, however, CFU/mL recovered from acidified EG media were decreased by ∼1000-fold for TW14359Δ*rpoN*Gln+ when compared to TW14359Δ*rpoN* (Fig.3C). Deletion of *glnA* in TW14359Δ*rpoN*Gln+ again restored survival in acid comparable to that of TW14359Δ*rpoN* (Fig.3C), suggesting that glutamine synthetase production plays an indirect role in EHEC acid resistance. Overexpression of *glnA* in TW14359Δ*rpoN* (strain TW14359Δ*rpoN*pRAM-9) similarly mitigated the acid resistance phenotype of TW14359Δ*rpoN* (Fig.3C), clearly demonstrating a role for *glnA* in the complete acid resistance phenotype of TW14359Δ*rpoN*. Adding to this, CFU/mL recovered from acidified EG increased by ≥100-fold in TW14359Δ*glnA* compared to TW14359. Consistent with qRT-PCR data on *ler* (Fig.3A), the number of microcolonies formed on HT-29 cells in TW14359Δ*rpoN*Gln+ was significantly reduced when compared to TW14359 (*P* < 0.05), but did not differ from TW14359Δ*rpoN* or TW14359Δ*rpoN*Δ*glnA*Gln+, collectively suggesting that changes in glutamine availability has no effect on *σ*^N^-dependent LEE expression and adherence to intestinal cells (Fig.3D).

### Acetyl phosphate stimulates the NtrC-*σ*^N^-pathway controlling GDAR and LEE expression

When *E. coli* is cultivated in media without ammonia, intracellular levels of glutamine are low, culminating in the phosphorylation and activation of NtrC by sensor kinase NtrB and NtrC-*σ*^N^-dependent transcription. It was thus suspected that the absence of ammonia in DMEM may prompt NtrC-*σ*^N^-dependent transcription of *flhDC*, activating the pathway for GDAR and LEE regulation, and that supplementation of DMEM with ammonia would offset this effect. If so, ammonia would be expected to stimulate *gad* gene expression and repress the LEE in TW14359, but to have no effect in the TW14359Δ*rpoN* and TW14359Δ*ntrC* backgrounds.

**Figure 4:**
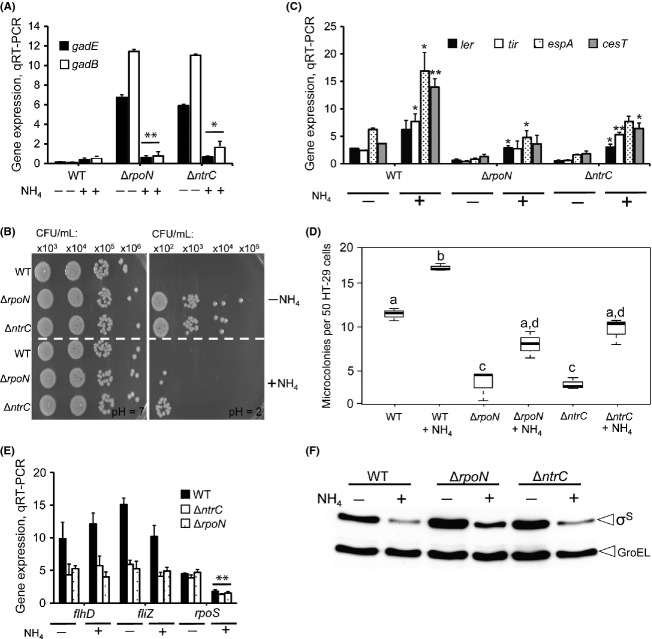
Role for ammonium in the NtrC-*σ*^N^-dependent pathway controlling GDAR and the LEE. (A) Expression levels for *gadE* (filled) and *gadB* (empty) without (−) and with (+) the addition of NH_4_Cl plotted for wild-type and derivative strains; asterisks denote significant difference between treatments by *t*-test (*P < 0.05, **P < 0.01, n≥3). (B) Representative colony-forming units (CFU/mL) on LBA for wild-type and derivative strains grown without (−NH_4_) or with (+NH_4_) NH_4_Cl added to DMEM and following 1-h challenge in EG media (pH 7 vs. pH 2). (C) Expression levels for *ler* (black), *tir* (white), *espA* (stippled), and *cesT* (gray) for wild-type and derivative strains grown without (−NH_3_) or with (+NH_3_) NH_4_Cl added to DMEM. (D) Counts for microcolonies on HT-29 cells plotted for wild-type and mutant derivative strains grown without (−NH_4_) or with (+NH_4_) NH_4_Cl. Boxplots are as described for Figure1D. (E) Expression levels of *flhDC*, *fliZ*, and *rpoS* plotted for wild type (black), Δ*ntrC* (white), and Δ*rpoN* (stippled). (F) Representative western blot for *σ*^S^ and GroEL (control) in wild type, Δ*rpoN*, and Δ*ntrC* grown without (−) or with (+) NH_4_Cl added to DMEM. For A, C, and E, asterisks denote significant differences between treatments by *t*-test (*P < 0.05, **P < 0.01, *n* ≥ 3). For D, plots that differ in lowercase letter differ significantly by Tukey's HSD following a significant *F*-test (*n* ≥ 3, *P* < 0.05). Error bars denote standard deviation. GDAR, glutamate-dependent acid resistance; LEE, locus of enterocyte effacement.

While the addition of ammonium chloride (2 g/L NH_4_Cl) was observed to slightly but not significantly increase GDAR gene (*gadE* and *gadB*) expression in TW14359, expression in TW14359Δ*ntrC* and TW14359Δ*rpoN* uniformly decreased (*P* < 0.05) (Fig.4A). Correspondingly, ammonium addition reduced CFU/mL recovered for TW14359Δ*ntrC* and TW14359Δ*rpoN* by ∼100- to 1000-fold but had no observable effect on CFU/mL recovered for TW14359 (Fig.4B). For the LEE, ammonium addition increased *ler*, *tir*, *espA*, and *cesT* expression in all backgrounds (Fig.4C) and correspondingly increased the number of microcolonies formed on HT-29 cells for all strains (*P* < 0.05). The same observations were made when substituting equimolar ammonium sulfate for ammonium chloride (data not shown). These results reveal that ammonium does in fact influence GDAR and LEE gene expression, but by a mechanism that is independent of *ntrC* and *rpoN*. In support of these data, the expression of pathway components (*gadE*, *ler*, *flhDC*, and *fliZ*) for control of GDAR and the LEE were not altered in a strain deleted for the NtrC cognate sensor kinase, *ntrB* (Fig. S1). Interestingly, growth in DMEM containing ammonium was observed to significantly reduce *rpoS* expression in TW14359, TW14359Δ*ntrC*, and TW14359Δ*rpoN* (*P* < 0.01), while having no impact on *flhDC* or *fliZ* expression in these backgrounds (Fig.4E). This reduction in *rpoS* transcript levels correlated with a reduction in *σ*^S^ levels in all backgrounds with ammonium, however, *σ*^S^ levels were not as strongly reduced in TW14359Δ*rpoN* when compared to TW14359 or TW14359Δ*ntrC* (Fig.4F).

**Figure 5:**
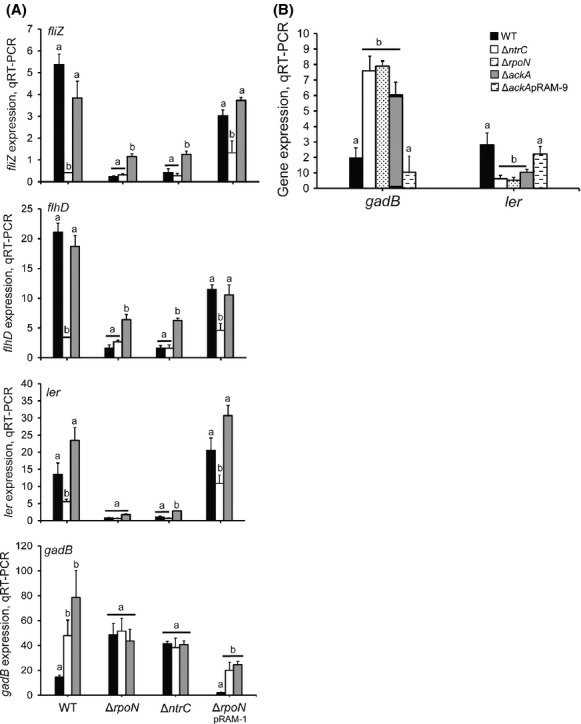
The effect of acetyl∼P availability on the expression of essential components for *σ*^N^-dependent regulation of GDAR and the LEE. (A) Expression levels of genes in order from bottom to top: *gadE*, *ler*, *flhD*, and *fliZ* plotted for wild-type and derivative strains grown in MOPS with glucose (black), glycerol (white), or glycerol and acetate (gray). (B) *gadE* and *ler* expression levels plotted for wild-type (black), Δ*ntrC* (white), Δ*rpoN* (stippled), Δ*ackA* (gray), and *ackA* complement strain Δ*ackA*pRAM-8 (hatched). Plots that differ in lowercase letter for each strain (A) or gene (B) differ significantly by Tukey's HSD following a significant *F*-test (*n* ≥ 3, *P* < 0.05). Error bars denote standard deviation. GDAR, glutamate-dependent acid resistance; LEE, locus of enterocyte effacement.

Feng et al. (1992) demonstrated phosphotransfer to, and activation of, NtrC in *E. coli* by the small molecule phosphodonor acetyl phosphate (acetyl∼P). Acetyl∼P readily accumulates during growth on glucose or in the presence of excess acetate, but not during growth on glycerol (McCleary and Stock 1994; Wolfe 2005). It was thus of interest to determine the effect of glucose and acetyl∼P availability on NtrC-*σ*^N^-dependent control of pathway components for the regulation of GDAR and the LEE. During growth in MOPS media containing glucose (2 g/L) and NH_4_Cl (1 g/L) (OD_600_ = 0.5), the expression of *flhDC*, *fliZ*, and *ler* was decreased and *gadB* increased in TW14359Δ*ntrC* and TW14359Δ*rpoN* when compared to TW14359 (*P* < 0.05) (Fig.5A), similar to that observed during growth in DMEM media (Figs.1A, B and 2A). Substituting 0.2% (v/v) glycerol for glucose as the sole carbon source reduced *flhDC*, *fliZ*, and *ler* expression in TW14359 and *rpoN* complement strain TW14359Δ*rpoN*pRAM-1 (*P* < 0.05), but not in TW14359Δ*ntrC* and TW14359Δ*rpoN* (Fig.5A). Likewise, glycerol substitution increased *gadB* expression in TW14359 and TW14359Δ*rpoN*pRAM-1 (*P* < 0.05), but not in TW14359Δ*ntrC* and TW14359Δ*rpoN*. The addition of sodium acetate (2 g/L) to glycerol treatments restored *flhDC*, *fliZ*, and *ler* expression to levels observed for glucose in TW14359, however, *gadB* expression was slightly but not significantly increased when compared to glycerol treatments (Fig.5A). In TW14359Δ*ntrC* and TW14359Δ*rpoN*, acetate was still observed to generally increase *fliZ*, *flhDC*, and *ler* expression, yet had no impact on *gadB* expression in these backgrounds, which may reflect a more generalized, *ntrC*- and *rpoN*-independent effect of acetate on the expression of these genes. To further examine the effect of acetate and acetyl∼P availability on this regulatory pathway, *gadB* and *ler* expressions were measured in a strain null for acetate kinase (*ackA*), the product of which catalyzes the interconversion of acetate to acetyl∼P (Rose et al. 1954). In TW14359Δ*ntrC*, TW14359Δ*rpoN*, and TW14359Δ*ackA*, *gadB* expression was significantly and uniformly increased when compared to TW14359 (*P* < 0.01) (Fig.5B). Complementation with *ackA* (strain TW14359Δ*ackA*pRAM-8) restored *gadB* expression to wild-type levels. For *ler*, expression was similarly reduced in TW14359Δ*ackA*, TW14359Δ*ntrC*, and TW14359Δ*rpoN* when compared to TW14359 and TW14359Δ*ackA*pRAM-8 (*P* < 0.05). Together, these data provide evidence that regulation of GDAR and the LEE by NtrC-*σ*^N^ is insensitive to changes in nitrogen availability (i.e., glutamine/ammonium), but instead is influenced by the availability of acetyl∼P.

## Discussion

In the present study, NtrC and *σ*^N^ have been shown to positively regulate the expression of *crl* and *fliZ*, the products of which control the activity of *σ*^S^. It is predicted that of the two, only FliZ is a required component of the *σ*^N^ pathway controlling *σ*^S^, GDAR, and the LEE. What impact *crl* upregulation in TW14359Δ*rpoN* has on *σ*^S^, if any, is as yet unclear. Crl and FliZ play antagonistic roles in the regulation of *σ*^S^. Crl directly binds *σ*^S^ facilitating interaction with RNA polymerase and holoenzyme (E*σ*^S^) formation (Bougdour et al. 2004), whereas FliZ acts downstream of E*σ*^S^ formation, binding to the -10 box of *σ*^S^ promoters (Pesavento and Hengge 2012) precluding promoter recognition by E*σ*^S^. Thus, FliZ may be dominant to Crl in *σ*^N^-directed control of *σ*^S^ activity. Alternatively, Crl reduces *σ*^S^ stability in an RssB-dependent manner during all stages of growth (Typas et al. 2007). It is therefore plausible that the increased stability of *σ*^S^ in *rpoN* null backgrounds (Dong et al. 2011; Mitra et al. 2012) results from reduced *crl* expression. This is consistent with the observation that in TW14359Δ*rpoN* the GDAR and LEE expression phenotype cannot be reproduced by increasing *σ*^S^ stability alone (Mitra et al. 2012).

**Figure 6:**
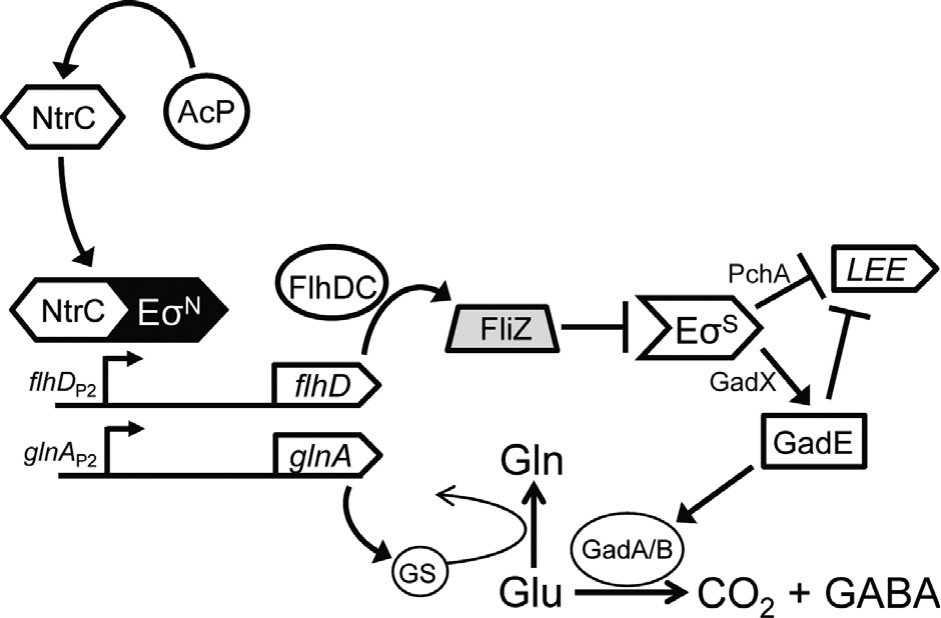
Model predicting NtrC-*σ*^N^-dependent regulation of GDAR and the LEE. During exponential growth in DMEM (a nitrogen-limiting media), NtrC activates transcription from *σ*^N^-dependent promoters for *flhD* and *glnA*. FlhDC (regulator of flagellar biosynthesis) directly activates *fliZ*, the product of which reduces the activity of *σ*^S^-RNAP (E*σ*^S^) holoenzyme. *σ*^S^ indirectly downregulates LEE expression by repressing the LEE activator *pchA* by an unknown mechanism, while upregulating the GDAR activator *gadE* through increased transcription of *gadX*. GadE has also been shown to directly repress transcription of *ler*. The upregulation of *glnA* (encoding glutamine synthetase, GS) increases the conversion of extant glutamate (Glu) to glutamine (Gln), thus depleting the substrate for GDAR system decarboxylases (GadA/GadB) and the potential for proton scavenging and acid detoxification. Acetyl∼P (AcP) is a noncognate phosphodonor that can activate NtrC-dependent transcription from *σ*^N^ promoters for *flhD* and *glnA*. The model is an amalgam of experimental observations inferred from this and previous studies (Reitzer et al. 1989; Feng et al. 1992; Tomoyasu et al. 2005; Kailasan Vanaja et al. 2009; Zhao et al. 2010; Lee et al. 2011; Pesavento and Hengge 2012; Branchu et al. 2014). See the text for further details. GDAR, glutamate-dependent acid resistance; LEE, locus of enterocyte effacement.

The transcription of *fliZ* is largely determined by FlhDC, a global regulator of motility genes (Francez-Charlot et al. 2003). FlhD forms a heterodimer with FlhC, directly activating transcription of the *fliAZY* operon from the *σ*^70^-dependent promoter *fliA*_P_. This study determined that *flhDC* was required for *σ*^N^-directed regulation of GDAR and LEE genes in a manner that was dependent on an intact *fliZ*. Based on our results, it is predicted that NtrC and *σ*^N^ directly activate transcription of *flhDC* during exponential growth in DMEM (4 g/L glucose, with no NH_4_) requiring the putative *σ*^N^-promoter *flhD*_P2_, and a newly identified NtrC box at positions 2481732–2487152 (Fig.6). This NtrC box is nearly identical to the predicted NtrC consensus (Ferro-Luzzi Ames and Nikaido 1985), differing by a single nucleotide in the dyad repeat region. Upregulation of FlhDC leads to increased transcription of *fliZ* (Francez-Charlot et al. 2003), the product of which decreases the activity of *σ*^S^ (Pesavento and Hengge 2012) (Fig.6). This suggests that during exponential growth NtrC-*σ*^N^ keep the activity of extant *σ*^S^ in check by increasing FlhDC-dependent transcription of *fliZ*. One consequence of this reduced *σ*^S^ activity in EHEC is an increase in LEE expression (Riordan et al. 2010) and correspondingly, increased in vitro microcolony formation. This could occur by at least two discrete mechanisms: by upregulation of *pchA* or through the downregulation of *gadE* (Fig.6). PchA is a LEE activator that is negatively regulated by *σ*^S^ (Iyoda and Watanabe 2005), whereas GadE represses the LEE and is activated by *σ*^S^ through upregulation of *gadX* (Ma et al. 2003). While the involvement of PchA in this pathway cannot be ruled out, only *gadE* and *gadX* expressions are significantly altered in the *rpoN* null background (Riordan et al. 2010). Even though FlhDC has been shown to effect adherence in *E. coli*, until now, the association has been negative. Leatham et al. (2005) reported that the deletion of *flhDC* in *E. coli* K-12 increased colonization of a mouse, while constitutive expression of *flhDC* in another study, reduced adherence of EHEC to HeLa cells (Iyoda et al. 2006). As the former study is in the K-12 MG1655 background, the effect of *flhDC* on colonization is clearly LEE independent. For EHEC, however, *flhDC* and the LEE are known to be inversely regulated; expression of LEE-encoded GrlA downregulates *flhDC* and motility in a manner dependent on RcsB, a response regulator of the Rcs phosphorelay system (Iyoda et al. 2006; Morgan et al. 2013). Perhaps FlhDC is used by *σ*^N^ to initiate transcription of the LEE, and then is repressed as GrlA accumulates as part of a GrlA-RcsB feedback loop initiating intimate adherence. This would be consistent with the transience and growth-phase dependence of *σ*^N^-dependent regulation of the LEE (Mitra et al. 2012).

By reducing the activity of *σ*^S^, *σ*^N^ also helps to maintain a low level of GDAR gene expression during exponential growth (Fig.6). However, unlike *σ*^N^-dependent LEE regulation, it is predicted that full expression of the GDAR phenotype in *rpoN* null strains is a consequence of two discrete but concurrent mechanisms. One requires *σ*^S^ for the activation of GDAR system genes (*gad* genes), the products of which confer acid resistance by a proton-scavenging mechanism involving the decarboxylation (GadA/GadB decarboxylases) and subsequent protonation of glutamate to yield *γ*-amino butyric acid (GABA) (Fig.6). In the absence of glutamate, GDAR is defective in protecting *E. coli* from acid stress (reviewed in Foster 2004). It is this cellular glutamate that is the source of the corresponding mechanism. Specifically, under nitrogen-limiting conditions (ex. growth in DMEM), NtrC-*σ*^N^ activate transcription of glutamine synthetase (*glnA*), which catalyzes the conversion of glutamate (Glu) to glutamine (Gln) (Fig.6). Strains null for *rpoN* or *ntrC* are therefore unable to activate *glnA* in response to reduced nitrogen availability, leading to glutamate accumulation and auxotrophy for glutamine. These strains are thus characterized by elevated levels of both the components (i.e., *gadE*, *gadA/B*, *gadC*) and substrate (glutamate) for GDAR. This mechanistic duality is reflected in the observation that neither *fliZ* nor *glnA* deletion can fully recapitulate the GDAR phenotype of an *rpoN* null background. Since as many as 60% of *σ*^N^-regulated genes have been shown to be antagonistically controlled by *σ*^S^ in *E. coli* (Dong et al. 2011), the interplay of these sigma factors likely has a more global impact on virulence, fitness, and metabolism than simply control of GDAR and the LEE.

The precise activating signal for NtrC-*σ*^N^-dependent regulation of GDAR and the LEE is as yet unknown. Phosphorylation and activation of NtrC is sensitive to changes in the intracellular levels of glutamine. When *E. coli* is grown in the absence of ammonium, glutamine levels are low, signaling the phosphorylation of NtrC by its cognate sensor kinase NtrB, and NtrC-dependent activation of *σ*^N^ promoters for nitrogen assimilation (Reitzer 2003). Although the addition of ammonium to DMEM did have a significant impact on GDAR and LEE expression, it did so independently of *ntrC* and *rpoN*. This effect of ammonium on the expression of *E. coli* colonizing factors has been formerly observed in EPEC, as well as for enterotoxigenic *E. coli* (ETEC). In EPEC, ammonium reduces expression of the bundle-forming pilus genes *bfpA* and *bfpT*, and reduces T3S-secretion of the EspA, EspB, and EspC translocon proteins (Puente et al. 1996; Kenny et al. 1997; Martinez-Laguna et al. 1999). For ETEC, ammonium increased expression of the 987P fimbria genes *fasH* and *fasA* (Edwards and Schifferli 1997). Changes in EPEC and ETEC colonizing factor expression in response to ammonium correlate with differences in tissue tropism and reflect the availability of ammonium in the intestine; its concentration gradually increases toward the distal small intestine (Toskes 1993; Edwards and Schifferli 1997; Martinez-Laguna et al. 1999). This natural gradient of intestinal ammonium may have a significant influence on the decision for colonization in all *E. coli*. However for EPEC, repression of *bfp* was shown to require a *trans-*acting factor that was absent, or present, but not functional in *E. coli* K-12 (Martinez-Laguna et al. 1999). How the ammonium signal is communicated to GDAR in EHEC and to the LEE in EHEC and EPEC requires further study.

Based on the findings of this study, it is proposed that NtrC is autophosphorylated by a noncognate phosphodonor in the *σ*^N^ pathway controlling GDAR and the LEE. Acetyl∼P is a plausible candidate (Fig.6), as it is a known NtrC phosphodonor (Feng et al. 1992; Atkinson and Ninfa 1998), and experimental alteration of acetyl∼P levels by substituting either glycerol or glycerol and acetate for glucose, or by the deletion of acetate kinase (*ackA*), altered the expression of pathway components for regulation of GDAR and the LEE in a manner dependent on *rpoN* and *ntrC*. Requirement for acetyl∼P is consistent with the growth-phase dependency of *σ*^N^ for GDAR and LEE regulation. The cellular pool of acetyl∼P during growth with glucose peaks during exponential phase, and drops off precipitously during transition into stationary phase (Takamura and Nomura 1988; Pruss and Wolfe 1994). Correspondingly, control of *gad* and LEE genes by NtrC and *σ*^N^ is restricted to the mid-exponential phase of growth (Riordan et al. 2010; Mitra et al. 2012). Remarkably, acetyl∼P also serves as a phosphodonor for Rrp2, a *σ*^N^ EBP found in *B. burgdorferi* and required for activation of the *σ*^N^-*σ*^S^ pathway regulating virulence expression in this pathogen (Xu et al. 2010). Thus, the use of acetyl∼P for autophosphorylation of *σ*^N^ EBPs may be a phenomenon that is conserved across different species of bacteria. Why acetyl∼P would be used in place of the cognate sensor kinase NtrB in *E. coli* is not yet known. It has been formerly proposed that the phosphorylation of NtrC by acetyl∼P may be used to initiate transcription of Ntr genes during transition to a nitrogen poor environment, as cellular NtrB levels are very low when nitrogen is abundant (Feng et al. 1992). Yet, in this study *ntrB* was clearly dispensable for GDAR and LEE regulation when grown in nitrogen-limiting media containing glucose, suggesting that acetyl∼P alone is sufficient to activate this pathway. It remains to be determined if *ntrB* is required for GDAR and LEE regulation by NtrC-*σ*^N^ in nitrogen-limiting media lacking glucose. The broader significance of this finding is that acetyl∼P levels in *E. coli* are sensitive to many factors, including nutrients, temperature, anaerobiosis, and pH (Wolfe 2005), suggesting that it may be used by NtrC to communicate various environmental cues to *σ*^N^.

## Conflict of Interest

None declared.

## Supporting Information

Figure S1 Effect of *ntrB* deletion on the expression of genes for GDAR and LEE control. Gene expression levels plotted for wild type (black), Δ*rpoN* (white), Δ*ntrC* (hatched), and Δ*ntrB* (gray). Asterisks denote significant difference between wild-type and respective mutants by *t*-test (**P* < 0.05, ***P* < 0.01, *n* ≥ 3). Error bars denote standard deviation.

Figure S2 Growth curve for TW14359 and mutant derivative strains in DMEM. Optical density (OD_600_) is plotted for each strain as a function of time. Samples were taken every hour for 12 h. OD_600_ measurements differed by less than 5% for each time point and strain.
