# Supplementary figures and images for: σN-dependent control of acid resistance and the locus of enterocyte effacement in enterohemorrhagic Escherichia coli is activated by acetyl phosphate in a manner requiring flagellar regulator FlhDC and the σS antagonist FliZ
Source: Microbiologyopen. 2014 Jun 16;3(4):497–512. doi: 10.1002/mbo3.183 (PMC4287178; doi:10.1002/mbo3.183)

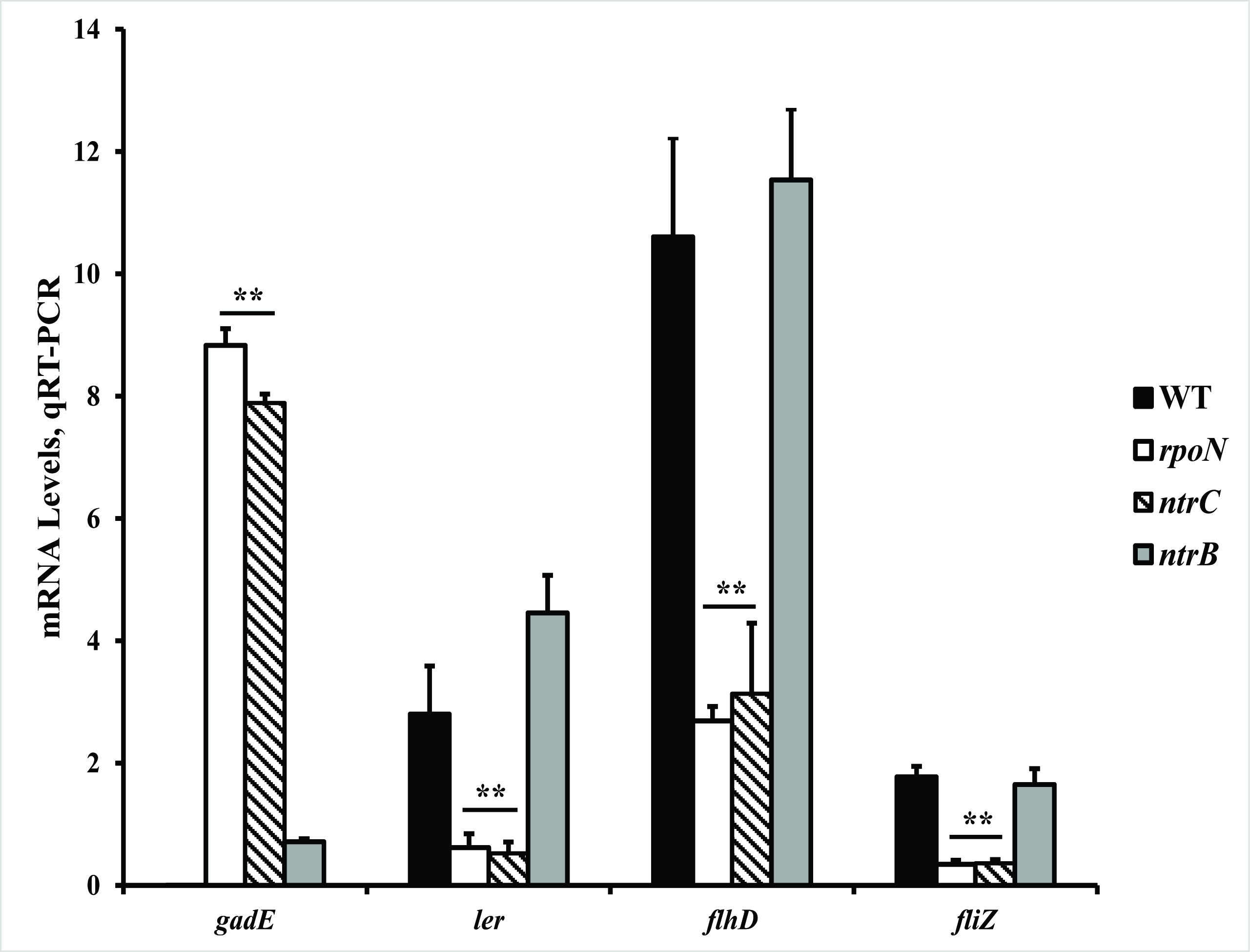

Supplement: Figure S1 — Effect of ntrB deletion on the expression of genes for GDAR and LEE control. Gene expression levels plotted for wild type (black), ΔrpoN (white), ΔntrC (hatched), and ΔntrB (gray). Asterisks denote significant difference between wild-type and respective mutants by t-test (*P < 0.05, **P < 0.01, n ≥ 3). Error bars denote standard deviation. [file mbo30003-0497-sd1.tif]

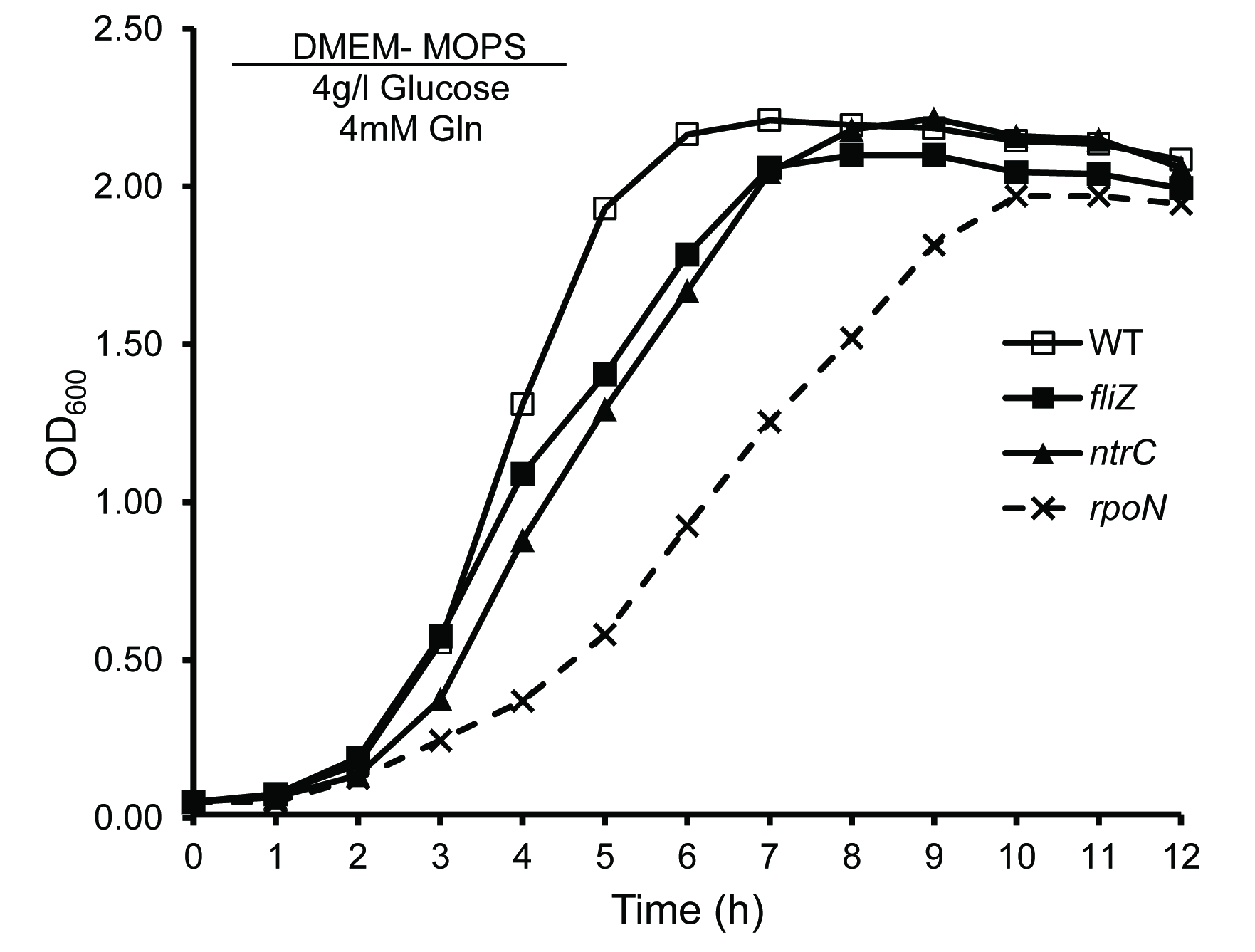

Supplement: Figure S2 — Growth curve for TW14359 and mutant derivative strains in DMEM. Optical density (OD600) is plotted for each strain as a function of time. Samples were taken every hour for 12 h. OD600 measurements differed by less than 5% for each time point and strain. [file mbo30003-0497-sd2.tif]
